# A neutralizing monoclonal antibody-based competitive ELISA for classical swine fever C-strain post–vaccination monitoring

**DOI:** 10.1186/s12917-020-2237-6

**Published:** 2020-01-14

**Authors:** Lihua Wang, Shijiang Mi, Rachel Madera, Llilianne Ganges, Manuel V. Borca, Jingqiang Ren, Chase Cunningham, Ada G. Cino-Ozuna, Hongwei Li, Changchun Tu, Wenjie Gong, Jishu Shi

**Affiliations:** 1 Department of Anatomy and Physiology, College of Veterinary Medicine, Kansas State University, Manhattan, KS USA; 2 Key Laboratory of Zoonoses Research, Ministry of Education, College of Veterinary Medicine, Jilin University, Changchun, Jilin China; 3 Institute of Military Veterinary, Academy of Military Medical Sciences, Changchun, Jilin, China; 4 OIE Reference Laboratory for Classical Swine Fever, IRTA-CReSA, Barcelona, Spain; 5 Plum Island Animal Disease Center, ARS, USDA, Orient Point, New York, USA; 6 Department of Diagnostic Medicine and Pathobiology, College of Veterinary Medicine, Kansas State University, Manhattan, KS USA; 7 School of Laboratory Medicine and Biotechnology, Southern Medical University, Guangzhou, Guangdong China

**Keywords:** Classical swine fever (CSF), C-strain, Monoclonal antibody, Competitive ELISA (cELISA), Virus neutralization test

## Abstract

**Background:**

Virus neutralization test (VNT) is widely used for serological survey of classical swine fever (CSF) and efficacy evaluation of CSF vaccines. However, VNT is a time consuming procedure that requires cell culture and live virus manipulation. C-strain CSF vaccine is the most frequently used vaccine for CSF control and prevention. In this study, we presented a neutralizing monoclonal antibody (mAb) based competitive enzyme-linked immunosorbent assay (cELISA) with the emphasis on the replacement of VNT for C-strain post–vaccination monitoring.

**Results:**

One monoclonal antibody (6B211) which has potent neutralizing activity against C-strain was generated. A novel cELISA was established and optimized based on the strategy that 6B211 can compete with C-strain induced neutralizing antibodies in pig serum to bind capture antigen C-strain E2. By testing C-strain VNT negative pig sera (*n* = 445) and C-strain VNT positive pig sera (*n* = 70), the 6B211 based cELISA showed 100% sensitivity (95% confidence interval: 94.87 to 100%) and 100% specificity (95% confidence interval: 100 to 100%). The C-strain antibody can be tested in pigs as early as 7 days post vaccination with the cELISA. By testing pig sera (*n* = 139) in parallel, the cELISA showed excellent agreement (Kappa = 0.957) with VNT. The inhibition rate of serum samples in the cELISA is highly correlated with their titers in VNT (*r*^2^ = 0.903, *p* < 0.001). In addition, intra- and inter-assays of the cELISA exhibited acceptable repeatability with low coefficient of variations (CVs).

**Conclusions:**

This novel cELISA demonstrated excellent agreement and high level correlation with VNT. It is a reliable tool for sero-monitoring of C-strain vaccination campaign because it is a rapid, simple, safe and cost effective assay that can be used to monitor vaccination-induced immune response at the population level.

## Background

Classical swine fever (CSF) is a highly contagious viral disease of swine, including wild (feral) and domestic pigs [1]. In many regions of the world, CSF is still endemic and is regarded as one of the major problems in pig industry [2]. CSF has the potential to cause devastating epidemics, particularly in countries free of the disease such as the United States [3, 4]. CSF control is primarily dependent on vaccination and the conventional Chinese vaccine (C-strain) is considered as one of the most effective vaccines because of its safety, quick protective immune response, crosss-protection against challenge of CSF viruses from different genotypes, and ability to be used for oral immunization of wild boars [2, 5–9].

The etiologic agent of CSF, classical swine fever virus (CSFV), is one member of the genus *Pestivirus* of the *Flaviviridae* family [10]. CSFV genome is comprised of a single large open reading frame (ORF) coding for a polyprotein encompassing all the viral proteins: four structural (C, E^rns^, E1, and E2) and eight nonstructural viral proteins (N^pro^, p7, NS2, NS3, NS4A, NS4B, NS5A, and NS5B) [11, 12]. The envelope glycoprotein E2 is responsible for eliciting neutralizing antibodies which are protective against virulent CSF virus and is also the target antigen for development of CSF vaccines, molecular and serological tests [13–16]. High sequence variability has been found in E2 protein among CSFVs. Based on the full-length E2 gene sequences, CSFV isolates could be divided into three genotypes (1, 2, and 3) as well as 11 subgenotypes (1.1–1.4, 2.1a, 2.1b, 2.1c, 2.1d, 2.2, 2.3, and 3.4) [17].

Virus neutralization test (VNT) is considered as the gold standard for serological monitoring and efficacy evaluation of CSF vaccines. However, it has several limitations including time-consuming, requirement of cell culture, the need for live virus manipulation, and relatively expensive [2, 18–21]. Here, we described a competitive ELISA (cELISA) developed with a neutralizing anti-E2 monoclonal antibody. This novel cELISA is a rapid, simple, safe and cost effective approach for detection of C-strain CSF vaccine-induced immune response.

## Results

### Generation of suitable capture antigen and competitive monoclonal antibodies

**Fig. 1:**
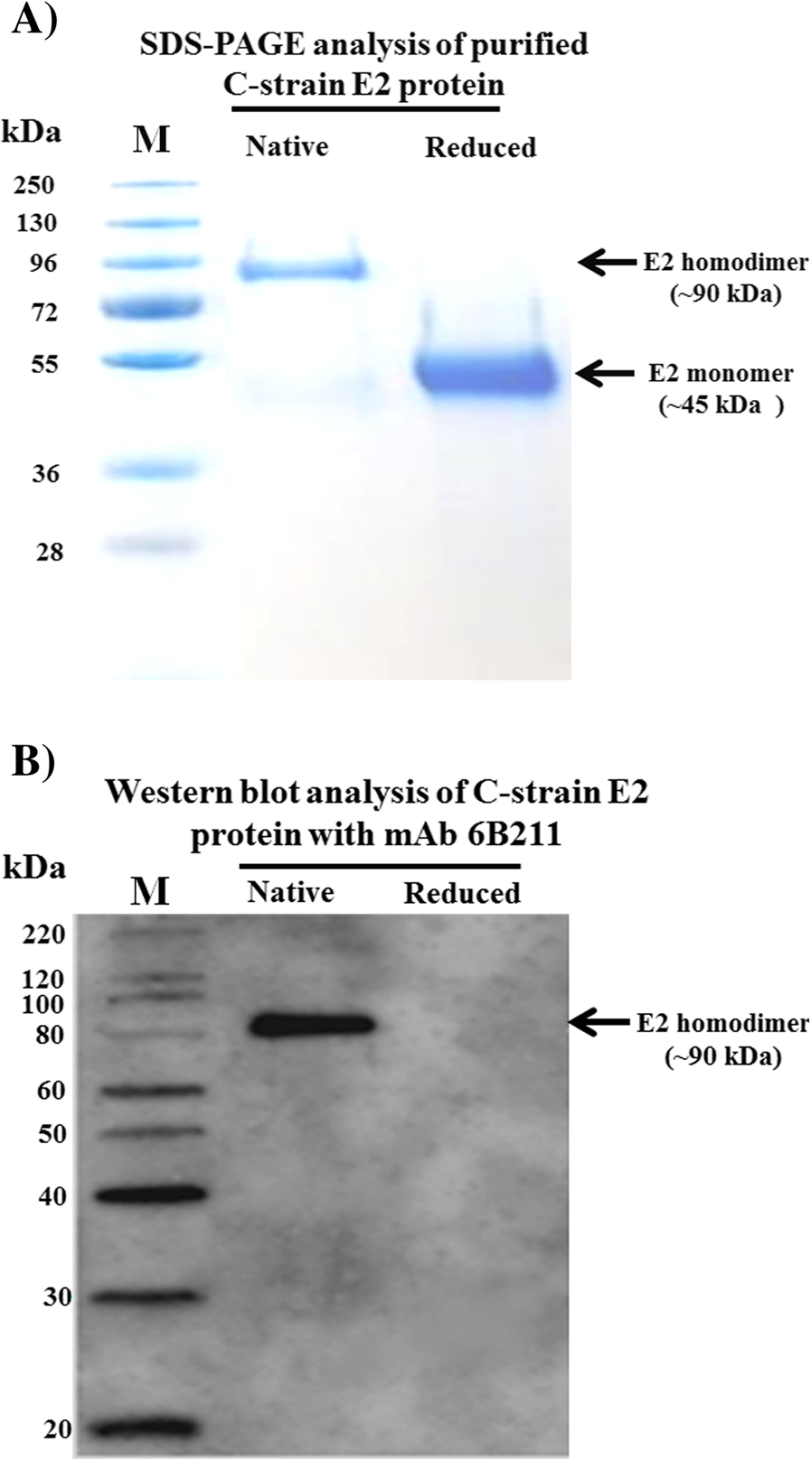
Analysis of purified C-strain E2 protein and monoclonal antibody 6B211. **a** Purified C-strain E2 protein mainly exists in its native dimeric conformation. After purification steps, the purified insect cell expressed C-strain E2 protein were treated without (Native) or with β-mercaptoethanol (Reduced) and separated by SDS-PAGE in a Mini-Protean TGX Gel (Bio-Rad, CA, USA); **b** 6B211 only react with the native C-strain proteins. Purified E2 proteins (native or reduced) were loaded on Mini-Protean TGX Gel. The proteins were then transferred to PVDF membrane and the membrane were blocked and incubated with 6B211

The envelope glycoprotein E2 of C-strain CSFV was successfully expressed in insect cells by using Bac-to-Bac® Baculovirus Expression System. The purified C-strain E2 protein mainly exists as homodimers (the native dimeric conformation) under non-reducing condition with a molecular weight of ~ 90 kDa (Fig. 1a).

**Fig. 2:**
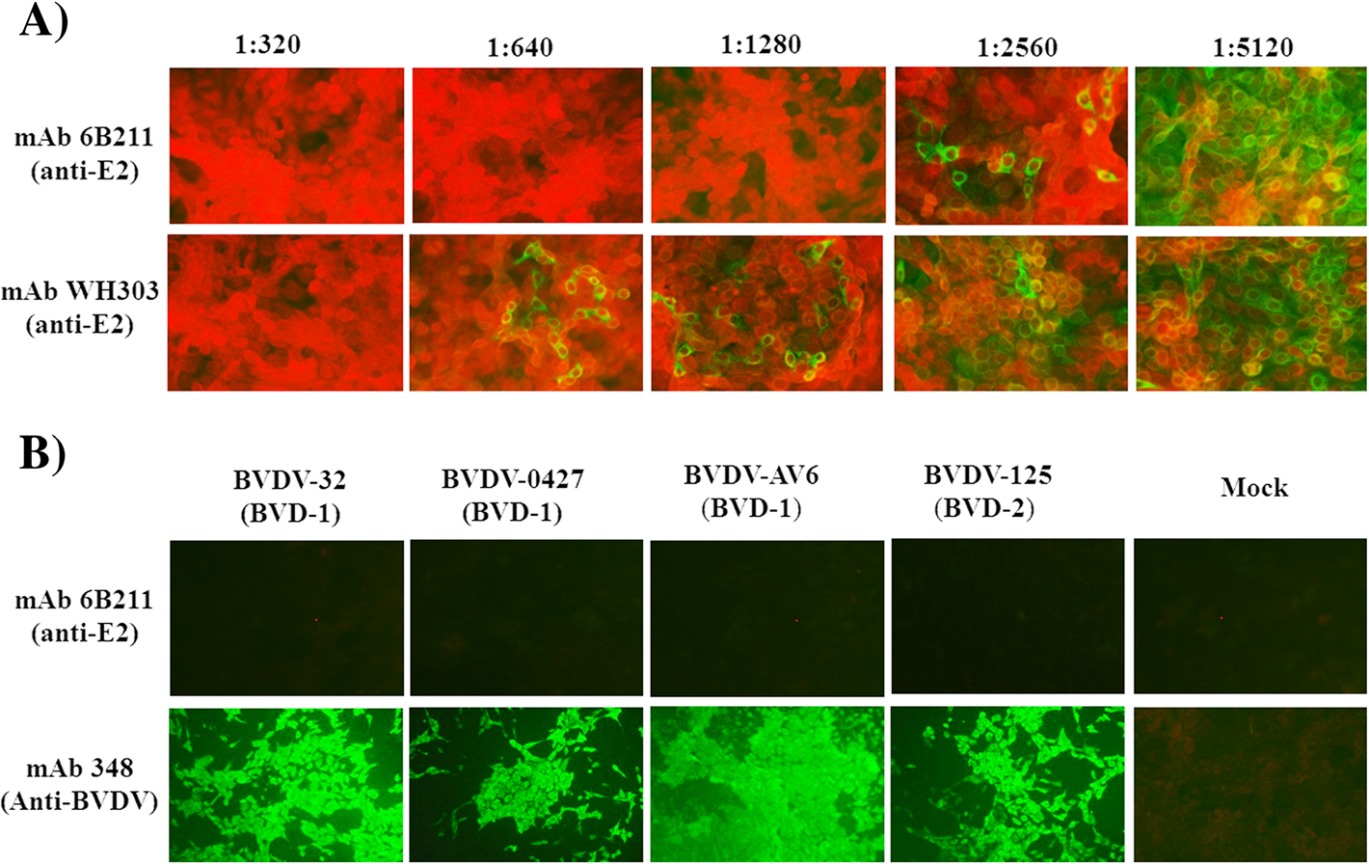
Neutralizing activity and cross-reaction testing of 6B211**. a** 6B211 has potent neutralizing activity against C-strain; ST cells were incubated with CSFV C-strain virus (100 TCID_50_) and two-fold serial dilutions (1:320 to 1:5120) of mAb 6B211 (1 mg/ml) or WH303 (1 mg/ml); 3 days post infection (DPI); no green fluorescent signal means 100% inhibition of C-strain virus; **b** 6B211 lacks cross-reactivity to BVDVs tested by IFA. Cells: MDBK; inoculated viruses: BVDV-32 (genotype 1, BVD-1), BVDV-0427 (BVD-1), BVDV-AV6 (BVD-1), and BVDV-125 (Genotype 2, BVD-2); 3 DPI; no green fluorescent signal means without reaction with BVDVs

To generate suitable mAb for the cELISA, purified C-strain E2 protein was used as immunogen for mAb production using Balb/c mice. All mice maintained good physical health and no adverse event happened during the experiments. Spleen cells from one mouse with the highest anti-E2 antibody titer were collected for fusion. One panel of more than 5 mAbs against C-strain E2 protein was generated. After assessment by VNT, mAb 6B211 (IgG1 and kappa chain) showed the most potent neutralizing activity against C-strain CSFV. 6B211 only react with homodimer of E2 protein and cannot recognize the reduced proteins, which indicate that it recognize the conformational epitope of C-strain E2 protein (Fig. 1b). The neutralization titer (neutralization doses 50%, ND_50_) of its purified supernatant (1 mg/ml with 1920 ND_50_) is much higher than that of the commercial neutralizing E2 monoclonal antibody WH303 (1 mg/ml with 480 ND_50_) (Fig. 2a). In addition, 6B211 lacks cross-reaction with other viruses in genus *Pestivirus* such as Bovine viral diarrhea virus (BVDV) (Fig. 2b).

### Establishment of competitive ELISA based on C-strain E2 protein and 6B211

**Fig. 3:**
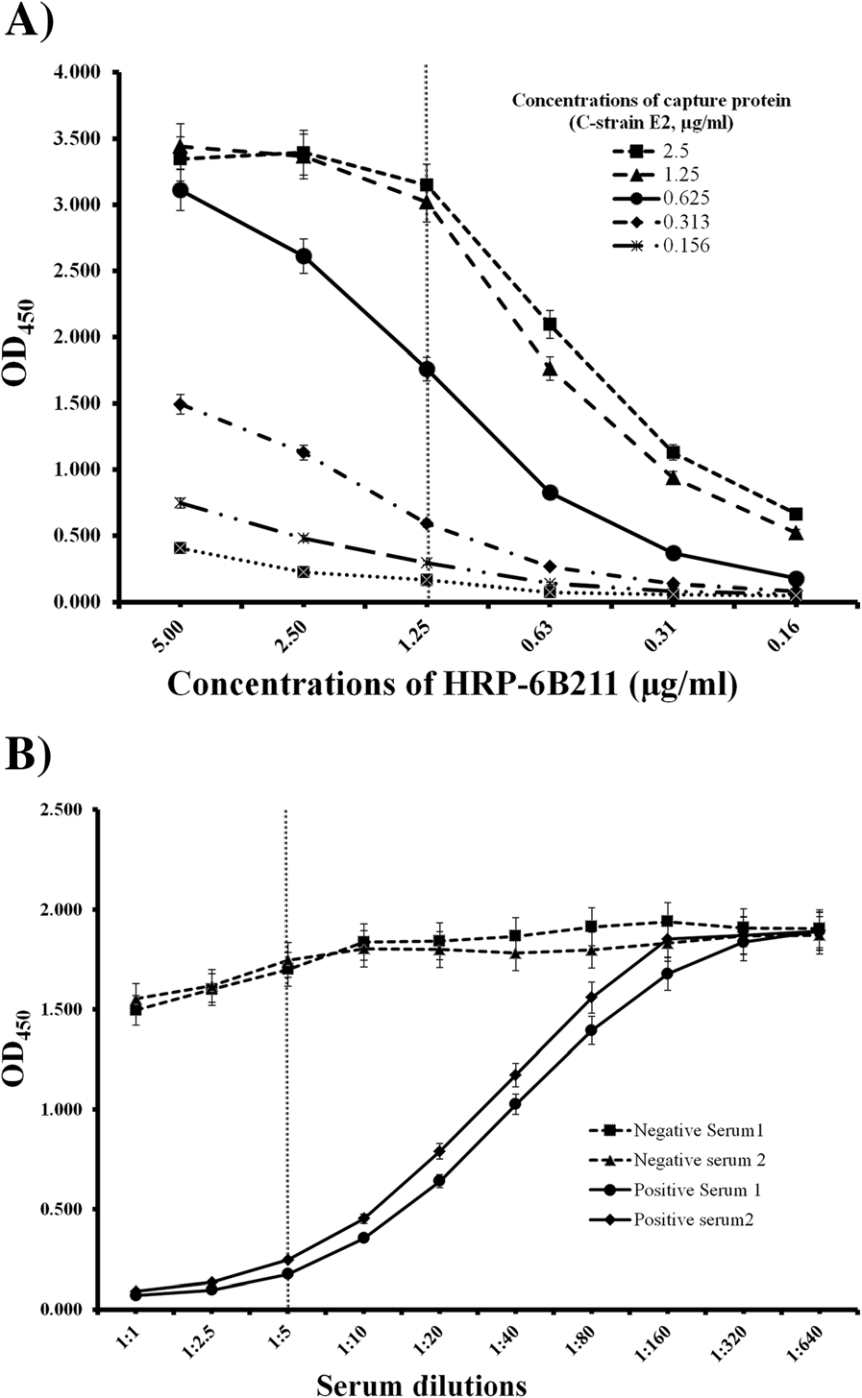
Determination of concentrations of capture antigen & competitive antibody, and dilution of serum. **a** Determination of optimal concentrations of capture protein (C-strain E2) and competitive antibody (HRP-6B211); the optimal concentration of capture protein (0.625 μg/ml) and HRP-6B211 (1.25 μg/ml) was chosen as it consistently produced an OD_450_ around 1.7 and at a point in the linear range that allowed optimal inhibition; **b** Determination of an optimal dilution of serum; To minimize the volume of serum required and background noise, serum dilution of 1:5 was chosen as the preferred dilution over lower dilutions that performed only slightly better. Data are expressed as the mean ± standard deviation from independently repeated experiments

Optimal concentrations of capture protein (C-strain E2) and competitive mAb (horseradish peroxidase conjugated 6B211, HRP-6B211) were determined by a systematic checkerboard procedure (Fig. 3a). A concentration of 0.625 μg/ml of C-strain E2 protein and a concentration of 1.25 μg/ml of HRP-6B211 were chosen as they consistently produced an OD_450_ value around 1.7 and at a point in the linear range in the standard curve. 2% fetal bovine serum (FBS) in phosphate buffered saline (PBS) containing 0.05% Tween 20 (PBST) was chosen as the optimal blocking buffer as it consistently outperformed other blocking solutions (non-fat dry milk and BSA) in terms of high signal to noise ratio. To minimize the volume of serum required and background noise, serum dilution of 1:5 was chosen as the preferred dilution over lower dilutions that performed only slightly better (Fig. 3b). These conditions were used in all subsequent cELISA experiments.

### Reproducibility of the cELISA

The reproducibility of the cELISA was determined by calculating the coefficient of variation (CV) of the percent of inhibition (PI) values by testing 20 C-strain VNT negative pig serum samples and 20 C-strain VNT positive pig serum samples. The intra-assay CVs of the C-strain VNT positive samples ranged from 0.02 to 1.68%. The inter-assay CVs of those same samples ranged from 1.02 to 8.66%. The intra-assay and inter-assay of the C-strain VNT negative samples also exhibited excellent repeatability, showing 0.01–0.35% and 0.66–4.29%, respectively (Table 1).

**Table 1:** Coefficient values of the samples tested by 6B211 based cELSIA

Pig sera	No. of sera tested	CV range (%)	
		Intra-assay	Inter-assay
C-strain VNT positive	20	0.02–1.68	1.02–8.66
C-strain VNT negative	20	0.01–0.35	0.66–4.29

### Comparison of 6B211 based cELISA with VNT

**Fig. 4:**
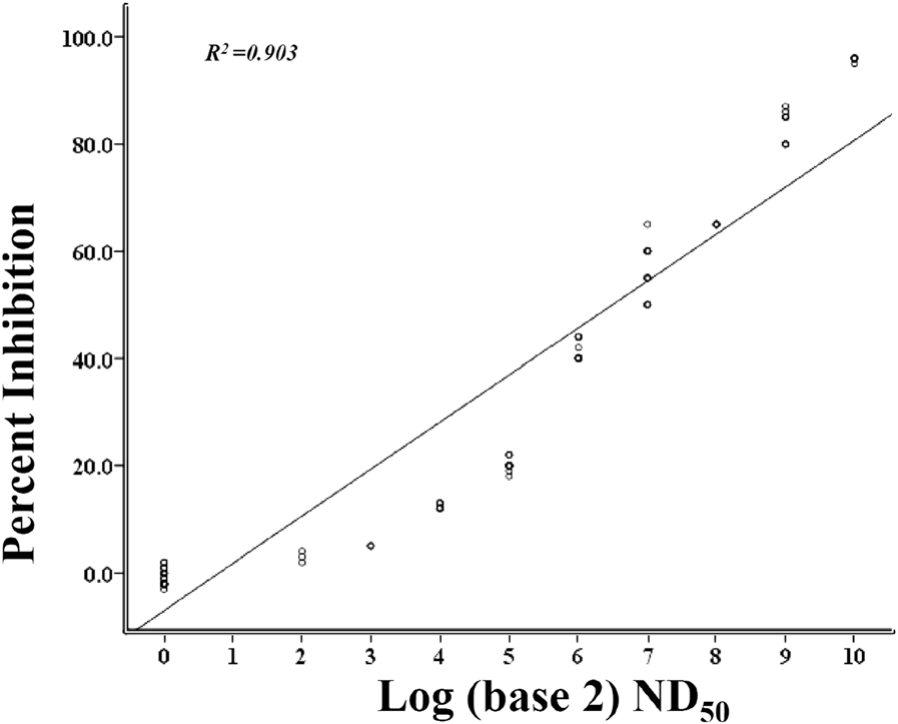
Correlation of inhibition rate of 6B211 based cELISA and titers of VNT against C-strain. Data represent the mean of independently repeated experiments

A total of 139 pig serum samples were tested using the established cELISA and VNT in parallel. Results (Table 2) showed that cELISA and VNT had an excellent agreement (Kappa = 0.957) in identifying positive (from pigs vaccinated with C-strain vaccine) and negative samples. McNemar’s test revealed that there was no significant difference in the results between the cELISA and VNT (*P* > 0.1). The Pearson correlation coefficient between the inhibition rate in 6B211 based cELISA and titers in VNT was calculated (Fig. 4) based on testing results of the 139 samples. The inhibition rate is highly correlated with VNT titers for these samples (*r*^2^ = 0.903, *p* < 0.001).

**Table 2:** Comparison of 6B211 based cELISA with VNT

	cELISA	VNT
Number positive	66	69
% Positive	47.5%	49.6%
Number negative	73	70
% Negative	52.5%	50.4%
Result agreement (Kappa)	0.957	
Significance	*P* > 0.1	

Note. Significance: *P* < 0.05 = tests are significantly different at the 95% confidence level

### Standardization of the cut-off value of 6B211 based cELISA

**Fig. 5:**
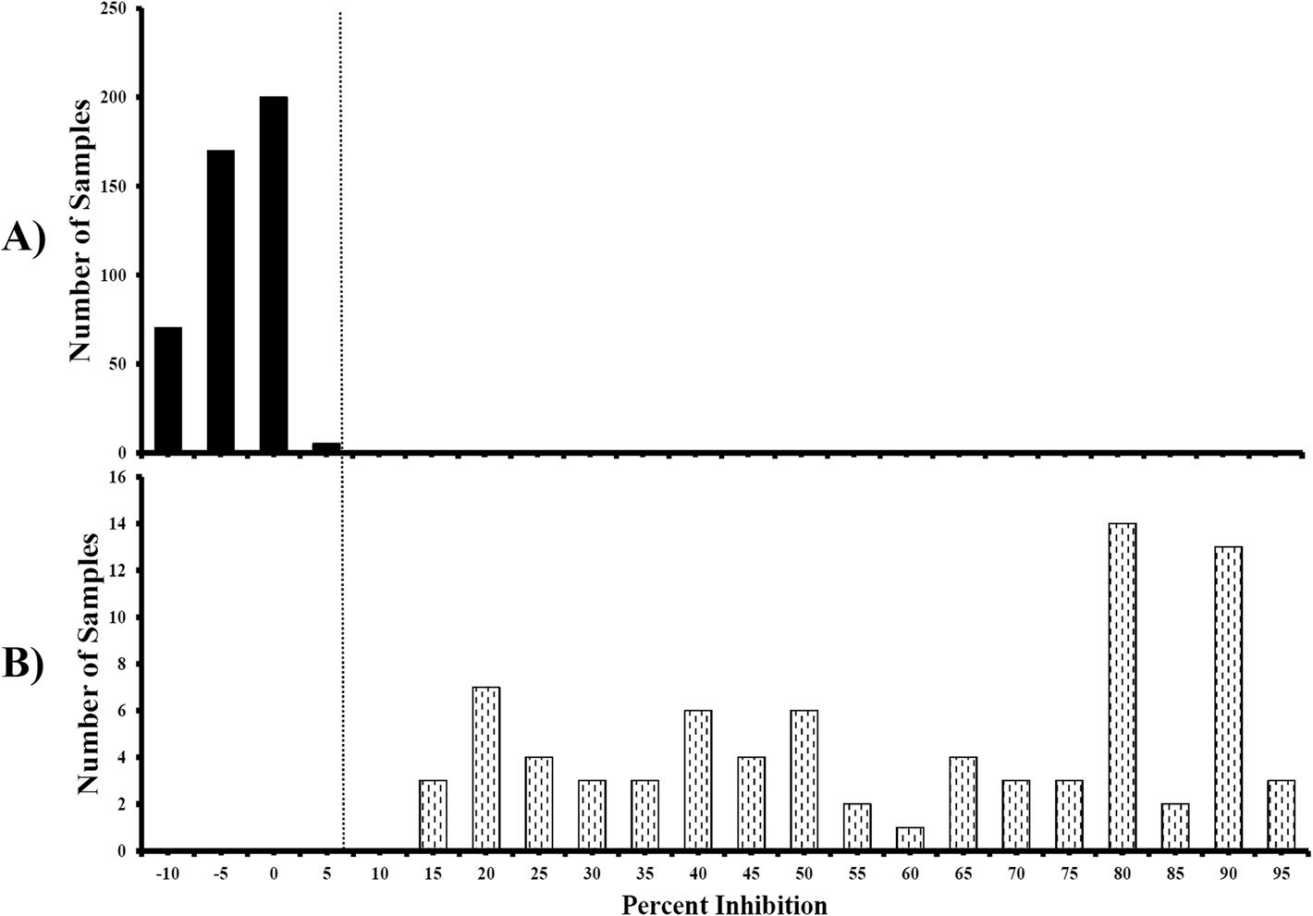
Standardization of the cut-off value of 6B211 based cELISA. **a** C-strain VNT negative serum samples (*n* = 445); **b** C-strain VNT positive serum samples (*n* = 70). The dotted line represents cut-off value of 6.19% inhibition when using the mean PI of negative sera plus three SD as the threshold

A total of 515 pig serum samples were used to standardize the cut-off value of the established cELISA. Among them, 445 samples were C-strain VNT negative and 70 samples were C-strain VNT positive (21 to 56 days post vaccination, DPV). After testing these samples by the established cELISA, distributions of the cELISA PI values showing the frequency of positive and negative samples are calculated and shown in Fig. 5. The mean PI value (x-axis) of the negative sera detected by cELISA was − 0.59%. When mean PI of negative sera plus two standard deviation (SD, 4.03%) was used as threshold, the sensitivity and specificity of the cELISA were 100% (95% confidence interval: 94.87 to 100%) and 98.43% (95% confidence interval: 96.79 to 99.37%), respectively. When mean PI of negative sera plus three SD (6.19%) was used as threshold, the sensitivity and specificity of the cELISA were 100% (95% confidence interval: 94.87 to 100%) and 100% (95% confidence interval: 100 to 100%), respectively.

### Kinetics of antibody response of pigs at different time intervals post-vaccination

**Fig. 6:**
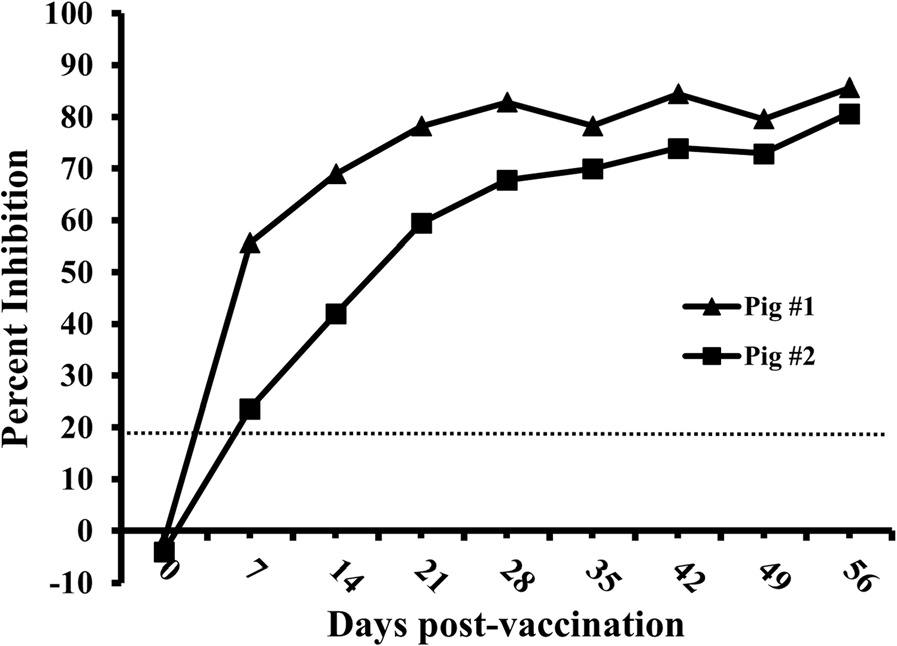
Kinetics of antibody responses of pigs tested by 6B211 based cELISA. Serum samples were derived from C-strain vaccinated pigs (*n* = 2) at every 7 days. The dotted line represents cut-off value: 19

Serial serum samples (0 to 56 DPV at every 7 days) derived from two pigs vaccinated with C-strain were tested by the established cELISA. The C-strain antibody could be tested in both pigs as early as 7 DPV with inhibition values 24 and 56%, respectively. Significant increase of inhibition was observed between 7 DPV and 28 DPV. The levels of antibody titer kept relatively stable from 28 DPV to 56 DPV with inhibition values ranging from 68 to 86% (Fig. 6).

## Discussion

In this study, we developed a neutralizing mAb based cELISA with the emphasis on the replacement of VNT for C-strain post–vaccination monitoring. Several monoclonal antibody-based cELISAs have been developed for detection and titration of antibodies against different viruses. One study developed a cELISA based on a mAb to a neutralizing epitope of hemagglutinin protein of *peste des petits ruminants virus* (PPRV). Efficacy of the cELISA compared very well with VNT having high relative specificity (98.4%) and sensitivity (92.4%). Findings suggest that the cELISA test developed can easily replace VNT for sero-surveillance, sero-monitoring, diagnosis from paired sera samples and end-point titration of PPRV antibodies [22]. Another study developed a cELISA for detecting antibodies against canine distemper virus (CDV) and phocine distemper virus (PDV) in sera from dogs and various species of marine mammals. The authors suggested that as cELISA proved to be nearly as sensitive and specific as the VNT while being simpler and more rapid, it would be an adequate screening test for CDV or PDV cases [23]. The glycoprotein E2 exposed on the outer surface of CSFV is the major immuno-protective antigen of C-strain vaccine that is responsible for inducing neutralizing antibodies and eliciting protective immunity against CSFV [13–20]. Thus, it is not surprising that E2 protein has been successfully used to develop ELISAs to measure anti-CSFV antibody response in pigs after vaccination [13–16]. In this study, the purified insect cell expressed C-strain envelope glycoprotein E2 was used as the capture antigen. The insect cell expressed E2 provides proper refolding, post-translational modification, and oligomerization [24], which guaranteed the immunodominant epitopes of E2 protein exposed in their native state.

The mAb 6B211 showed potent neutralizing activity against C-strain CSFV and bound to a specific conformational epitope on C-strain E2 protein (Fig. 1b & Fig. 2a). In addition, it showed very high sensitivity when tested by IFA (mouse ascites of 6B211 produced a strong positive fluorescence signal at dilution 1:16,000, data not shown). To test the hypothesis that neutralizing 6B211 can compete with C-strain induced neutralizing antibodies in pig serum to bind the capture antigen (C-strain E2), 445 C-strain VNT negative pig serum samples and 70 C-strain VNT positive pig serum samples were tested by the 6B211 based cELISA. The 6B211 based cELISA can effectively differentiate the C-strain VNT positive and C-strain VNT negative samples (Fig. 5), which indicate that 6B211 can compete with C-strain induced neutralizing antibodies from pigs and can be used as the competitive antibody for cELISA.

By testing the pig sera in parallel and using mean PI of negative sera plus three SD as the threshold, the sensitivity and specificity of the cELISA were 100% (95% confidence interval: 94.87 to 100%) and 100% (95% confidence interval: 100 to 100%), respectively. The C-strain antibody can be tested in pigs as early as 7 DPV with the cELISA. The excellent agreement (Kappa = 0.957, Table 2) between cELISA and VNT, and the high correlation VNT (*r*^2^ = 0.903) between inhibition rate in cELISA and VNT titers of tested samples indicate that the 6B211 based cELISA can replace the VNT for C-strain post–vaccination monitoring. In addition, the intra-assay CVs and the inter-assay CVs of the 6B211 based cELISA (Table 2) are lower than 10% when tested with the negative and E2 antibody positive pig serum samples, which indicate that the established cELISA is repeatable with acceptable variations.

## Conclusions

Through the aforementioned experiments and analysis, we concluded that the mAb 6B211 based cELISA showed excellent agreement and high correlation with the VNT. This cELISA is a reliable, rapid, simple, safe, and cost-effective tool for sero-monitoring of C-strain vaccination at a population level. We believe that the cELISA presented in this study could be used to assist in CSF control and eradication.

## Methods

### Animals

Five female Balb/c mice (6 weeks old) weighing between 22 and 25 g were purchased from Charles River Laboratories, Inc. Wilmington, MA, USA. The mice were fed with standard commercial diet and housed in a clean facility at the Kansas State University. Animal care and protocols were approved by Institutional Animal Care and Use Committee (IACUC#3517) at Kansas State University. All animal experiments were done under strict adherence to the IACUC protocols.

### Generation of monoclonal antibody to C-strain E2 protein

Expression and purification of C-strain E2 protein using a baculovirus expression system were performed as previously described [18]. The purified C-strain E2 protein was concentrated using Amicon Ultra Centrifugal Filters 30,000 NMWL (Millipore, Billerica, USA) and measured using BCA assay kit (Pierce, USA) according to the manufacture’s recommendations.

For mAb production, 50 μl (1 μg/μl) purified E2 protein plus equal volume of 2% Alhydrogel (Invitrogen, CA, USA) was used as an immunogen to inject each of the Balb/c mice via intraperitoneal injection. Three booster immunizations with same dose were conducted at 2 week intervals. Three days after the final booster injection, one mouse with the highest anti-E2 antibody titer was humanely euthanized using carbon dioxide (CO_2_) in euthanasia chambers and sprayed with 70% ethanol. Spleen cells were collected and fused with the mouse myeloma partner SP2/0-Ag14 (ATCC, MD, USA) by using polyethylene glycol 1500 (Boehringer Mannheim, IN, USA) at a ratio of 10:1. The hybridoma cells were maintained in RPMI1640 medium (Gibco, NY, USA) with 20% fetal bovine serum (FBS, Hyclone, UT, USA). Supernatants from growing hybridomas were screened by an ELISA for reactivity to E2 protein as previously described [18]. The positive hybridoma clones were subcloned three times by limiting dilution until monoclones were obtained. Characterization of these monoclonal antibodies will be published elsewhere. One mAb, designated 6B211 was used in this study. Its isotype was classified with an antibody-isotyping kit (Roche Diagnostics Corporation, IN, USA). The reaction of 6B211 with native and β-mercaptoethanol treated C-strain E2 protein was analyzed by western blot as we described previously [18].

### Indirect fluorescent antibody assay (IFA) test

The reactivity of mAb with different BVDVs was tested by IFA as described previously [25]. Briefly, MDBK cells (bovine kidney cells, ATCC, CCL22) grown in 96-well plate were infected with BVDVs (BVDV-32 strain, genotype 1; BVDV-0427 strain, genotype 1; BVDV-AV6 strain, genotype 1; BVDV-125 strain, genotype 2) at a multiplicity of infection (MOI) of 0.1 for 3 days. Cells were fixed in cold acetone and washed two times with PBST. Supernatant of hybridomas (1:50 diluted) was added and plates were incubated at 37 °C for 1 h (hr). Plates were washed three times with PBST and Alexa Fluor 488 goat anti-mouse IgG (H + L) (Life Technologies, MA, USA) was added at 1:200 dilution to each well and incubated at 37 °C for 1 h. Finally, the plate was washed three times with PBST and examined under a fluorescence microscope.

### Neutralizing antibody test

Purified mAbs 6B211 (1 mg/ml) and WH303 (1 mg/ml, Animal and Plant Health Laboratories Agency, Wey Bridge, United Kingdom) were first diluted five-fold and then serially diluted two-fold. The diluted samples (in duplicate) were incubated with 100 TCID_50_ (50% tissue culture infective dose) of CSFV C-strain in DMEM with 10% FBS for 1 h at 37 °C. Residual virus infectivity was determined by adding 1.0 × 10^4^ ST cells to each well with serum-virus mixture in 96-well plate and incubated at 37 °C for 3 days. The cells were subjected to immunofluorescence staining with E2-specific mAb WH303 and Alexa Fluor 488 goat anti-mouse IgG (H + L) (Life Technologies, MA, USA). Neutralizing antibody titers (NAT) were expressed as the reciprocal of the highest dilution that caused 50% neutralization.

### Competitive enzyme-linked immunosorbent assay (cELISA)

The 6B211 was purified by HiTrap™ Protein G column (GE Healthcare Life Sciences, PA, USA) followed by conjugating with Horseradish Peroxidase (HRP) using EZ-Link™ Plus Activated Peroxidase (Thermo Scientific, NJ, USA) according to the manufacturer’s instruction. The HRP-6B211 was dialyzed with Slide-A-Lyzer Dialysis Cassettes (Thermo Scientific, NJ, USA) against PBS and stored in Pierce™ Peroxidase Conjugate Stabilizer (Thermo Scientific, NJ, USA).

The systematic checkerboard procedure was used to optimize the concentration of capture antigen and HRP-6B211. The optimal dilution of serum and blocking solution were determined experimentally. The established cELISA was performed in Corning® 96 Well Clear Flat Bottom Polystyrene High Bind Microplate (Corning, NY, USA). Briefly, plates were coated overnight with C-strain E2 (0.625 μg/ml, 100 μl/well) in PBS (without calcium and magnesium, pH 7.4, Thermo Scientific, NJ, USA) at 4 °C. After washing three times with PBST, the plates were blocked with blocking buffer by incubating at 37 °C for 1 h; after washing, 50 μl of diluted serum samples and 50 μl of diluted HRP-6B211 were added to each well and mixed well by pipetting. The plates were incubated at 37 °C for 1 h. After washing five times, 100 μl of room-temperature TMB Stabilized Chromogen (Invitrogen, CA, USA) were added and incubated at room temperature (RT) for 10 min; after adding 100 μl/well of 2 N Sulfuric Acid (Ricca Chemical Company, TX, USA), the absorbance at 450 nm were obtained using SpectraMAX microplate reader (Molecular Devices, CA, USA). The OD_450_ of the samples were converted to a percent inhibition (PI) value using the following formulation: PI (%) = (OD_450_ value of negative controls − OD_450_ value of sample)/OD_450_ value of negative controls × 100%.

The cut-off value that served as the threshold to separate VNT positive sera from VNT negative sera was determined by testing negative sera of unvaccinated pigs and VNT positive sera of C-strain or C-strain E2 subunit vaccinated pigs (21–56 DPV).

Serial derived serum samples after vaccination with C-strain from 0 to 56 DPV at every 7 days were used for testing the kinetics of antibody titers of pigs (*n* = 2) at different time intervals post-vaccination.

### Reproducibility and statistical analysis of the cELISA

Inter-assay and intra-assay reproducibility for the established cELISA was evaluated by testing CSFV antibody negative (*n* = 20) serum samples and C-strain VNT positive pig serum samples (*n* = 20). For the intra-assay reproducibility, each serum sample (in duplicate) was detected by the same batch of pre-coated ELISA plates. For the inter-assay reproducibility, each serum sample was detected by three batches of pre-coated ELISA plates. Sensitivity and specificity analysis were carried out by the web-based MedCalc statistical software (https://www.medcalc.org/calc/diagnostic_test.php). Statistical analysis of reproducibility was carried out by calculate the mean PI value and coefficient of variation (CV) of replications of each test. Statistical analysis of the degree of agreement (Kappa value) and correlation between the established cELISA and VNT were carried out by McNemar’s test and Pearson correlation coefficient analysis in SPSS Statistics for Windows, version 25.0 (IBM Crop, NY, USA). Differences were considered statistically significant when *p* < 0.05. Scatter plots was generated using the same program.

## Data Availability

The datasets for the current study are available from the corresponding author on reasonable request.

## Abbreviations

BVDV: Bovine viral diarrhea virus
CDV: Canine distemper virus
cELISA: Competitive enzyme-linked immunosorbent assay
CO_2_: Carbon dioxide
CSF: Classical swine fever
CSFV: Classical swine fever virus
CV: Coefficient of variation
DPI: Days post infection
DPV: Days post vaccination
FBS: Fetal bovine serum
hr.: Hour
HRP: Horseradish peroxidase
IFA: Indirect fluorescent antibody assay
mAb: Monoclonal antibody
MOI: Multiplicity of infection
ND_50_: Neutralization doses 50%
ORF: Open reading frame
PBS: Phosphate-buffered saline
PBST: Phosphate buffered saline containing 0.05% Tween 20
PDV: Phocine distemper virus
PI: Percent of inhibition
PPRV: Peste des petits ruminants virus
RT: Room temperature
SD: Standard deviation
TCID_50_: 50% tissue culture infective dose
VNT: Virus neutralization test
